# Bilateral Idiopathic Multifocal Retinal Pigment Epithelial Detachments: A Case Report and Brief Review of the Literature

**DOI:** 10.7759/cureus.86048

**Published:** 2025-06-15

**Authors:** Dimitrios Kalogeropoulos, Ahmed Ibraheem, Daniel Dragnev

**Affiliations:** 1 Ophthalmology, St Helens Hospital, Mersey and West Lancashire Teaching Hospitals NHS Trust, St Helens, GBR

**Keywords:** fundus examination, heidelberg retina tomograph, optical coherence tomography, retina, retina disease

## Abstract

Retinal pigment epithelial detachments (PEDs) result from a disruption at the junction between the basement membrane of the retinal pigment epithelium (RPE) and the innermost part of Bruch’s membrane. Serous retinal PEDs, whether singular or multiple, are frequently seen across a broad spectrum of ophthalmic disorders. Additionally, PEDs have been associated with various systemic conditions, including renal, hypertensive, endocrine, and hematologic disorders. Idiopathic PEDs, which occur without an associated underlying condition, are exceedingly rare, with only a few cases reported in the literature. Herein, we report a case of an asymptomatic and otherwise healthy individual with multiple idiopathic retinal PEDs, highlighting the importance of a comprehensive clinical examination and multimodal ophthalmic imaging.

## Introduction

Retinal pigment epithelial detachments (PEDs) occur from a disruption at the junction between the basement membrane of the retinal pigment epithelium (RPE) and the innermost part of Bruch’s membrane [1]. The resulting anatomical space from this process may become occupied by drusenoid deposits, fibrovascular tissue, serous exudate, blood, or a combination of these elements. Serous retinal PEDs, whether singular or multiple, are frequently seen across a broad spectrum of ophthalmic disorders, including age-related macular degeneration (AMD), central serous chorioretinopathy (CSCR), polypoidal choroidal vasculopathy (PCV), angioid streaks, Vogt-Koyanagi-Harada disease, and various hereditary chorioretinal degenerations [1, 2]. Additionally, PEDs have been reported in patients with renal disorders, malignant hypertension, systemic hypercortisolism, and acute leukaemia [3-6]. Idiopathic PEDs, which occur without an associated underlying condition, are exceedingly rare, with only a few cases reported in the literature. Herein, we report a case of an asymptomatic and otherwise healthy individual with multiple idiopathic retinal PEDs, highlighting the importance of a comprehensive clinical examination and multimodal ophthalmic imaging.

## Case presentation

**Figure 1:**
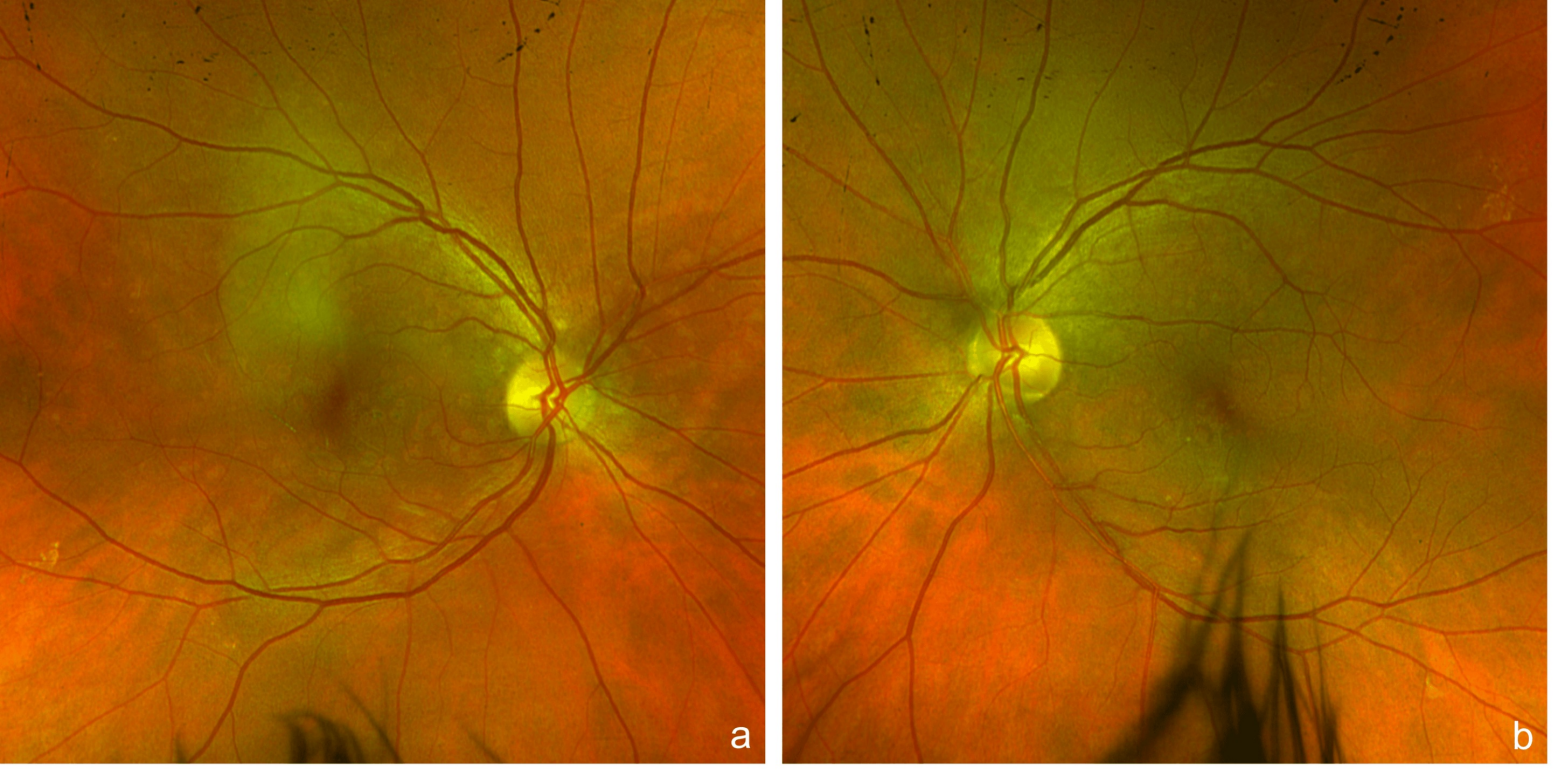
Optos widefield colour photos Optos widefield colour photos of the right (a) and left (b) eye, respectively, illustrating the distribution of multiple idiopathic pigment epithelial detachments (PEDs) across the posterior pole. The PEDs appear as numerous small yellowish lesions in the macular and peripapillary regions of both eyes.

**Figure 2:**
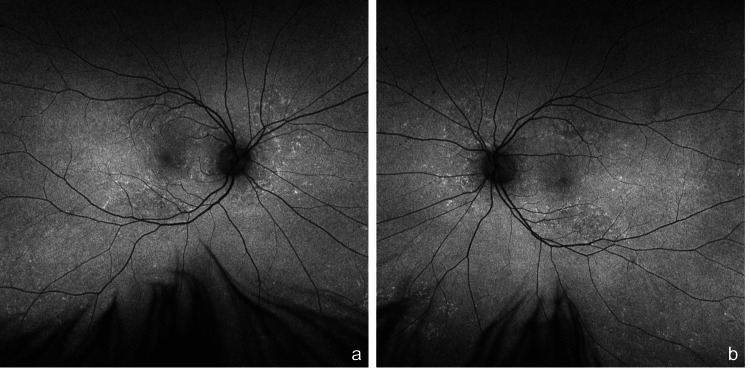
Optos widefield autofluorescence Optos widefield autofluorescence of the right (a) and left (b) eye, respectively, displaying multiple well-defined hyperautofluorescent lesions corresponding to the retinal pigment epithelial detachments.

**Figure 3:**
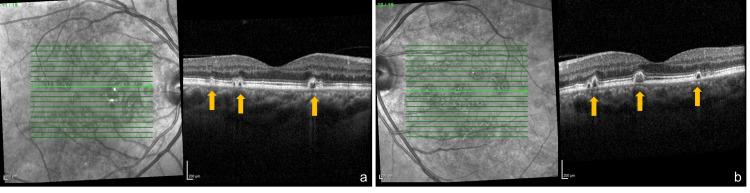
Spectral domain optical coherence tomography (SD-OCT) (Heidelberg Spectralis, Heidelberg Engineering, Germany) of the right (a) and the left (b) eye. OCT demonstrates multiple serous retinal pigment epithelial detachments appearing as dome-shaped elevations of the retinal pigment epithelium (yellow arrows). It is noteworthy that there is no evidence of associated subretinal fluid or choroidal neovascular membrane.

A 52-year-old asymptomatic White patient was referred to our department by her optician for further evaluation and treatment following a routine eye test, which demonstrated the presence of bilateral multiple PEDs. Her past ophthalmic, medical, and family histories were unexceptional. Snellen best-corrected visual acuity (BCVA) was 6/9, and the intraocular pressure (IOP) was normal, measuring 14 mmHg in both eyes. Anterior segment examination was unremarkable, but dilated fundoscopy revealed multiple bilateral serous PEDs. Ophthalmic imaging with Optos widefield photography (Figures 1a, 1b), autofluorescence (Figures 2a, 2b), and spectral domain optical coherence tomography (SD-OCT) (Figures 3a, 3b) was utilised to illustrate the distribution of PEDs, as well as the absence of subretinal or intraretinal fluid. Blood tests, performed to rule out any possible associated systemic conditions, were within normal limits, including full blood count, inflammatory markers (erythrocyte sedimentation rate (ESR) and C-reactive protein (CRP)), renal and liver function, ferritin, haemoglobin A1c, vitamin B12, folate, lipid profile, thyroid function, cortisol, and sex hormones. After a three-year follow-up, she remains clinically stable with no significant changes in the fundoscopic and imaging findings.

## Discussion

Although isolated serous retinal PEDs are frequently found in healthy asymptomatic individuals, the detection of idiopathic multiple retinal PEDs is relatively rare. Only a few cases have been reported in the existing literature. The exact pathogenetic mechanism remains unclear, but it has been suggested that extensive structural disruption in the adhesion between the RPE and Bruch’s membrane contributes to the formation of multiple PEDs [1].

Alhumaid et al. [6] reported two cases with bilateral idiopathic multifocal retinal PEDs in otherwise healthy young adults, underlining the importance of long-term follow-up to assess potential secondary complications and visual outcomes. Likewise, Dave et al. [7] described a case of a 51-year-old female and highlighted the need for a comprehensive systemic work-up and multimodal imaging. Interestingly, Chatzirallis et al. [8] demonstrated three cases that, although consistent with a diagnosis of multiple idiopathic PEDs, exhibited a miliary distribution spreading across the posterior pole. A key finding of this study is that the PEDs created a retinal appearance similar to crocodile skin on both fundus fluorescein (FFA) and indocyanine green angiography (ICGA). These features, observed in both dye tests, resemble the magnified cells seen in electron microscopy.

Idiopathic PEDs must be differentiated from drusen. They appear as smooth, dome-shaped elevations of the RPE with a hyporeflective sub-RPE space, suggesting clear fluid accumulation. On the other hand, drusen are illustrated as irregular, granular RPE elevations with hyperreflective internal content, typically without a well-defined dome shape. Moreover, drusen are often accompanied by retinal pigment mottling and overlying outer retinal disruption. Regarding idiopathic PEDs, the FFA in such cases shows late pooling without leakage. In contrast, when it comes to drusen, FFA displays early hyperfluorescence due to staining but no pooling or late increase in size. Additionally, the fluorescence pattern appears to be more punctate and scattered [8].

It has been hypothesised that idiopathic multiple serous retinal PEDs are a variant of CSCR. OCT shows a dome-shaped elevation of the RPE with a hyporeflective space beneath it. FFA demonstrates these PEDs with progressively increasing hyperfluorescence in the later frames, without any change in size, while ICGA reveals both hypofluorescent and hyperfluorescent lesions corresponding to the previously observed serous PEDs [9, 10]. Furthermore, a potential link between multiple PEDs and emotional stress, similar to what is seen in CSCR, has been reported [11]. In contrast with the AMD-associated PEDs, the formation of choroidal neovascular membranes (CNVM) is uncommon in PED linked to CSCR. Although the exact etiology and progression of multiple PEDs remain obscure, in case of suspicion, FFA and ICGA should be employed to exclude occult CNVM [12].

Although most cases are asymptomatic, complications like haemorrhagic PED [13], CNVM [14], and CSCR [15] may develop. While the diagnosis is primarily based on SD-OCT to confirm the serous nature of the PEDs, it is crucial to consider other potential aetiologies in the differential diagnosis. These include photoreceptor-RPE diastasis, which typically presents as multifocal CSCR [16]; non-Best multifocal vitelliform maculopathy, which features yellow lesions in the subretinal space; and Doyne’s honeycomb dystrophy, characterised by drusen with a temporal distribution and a family history [17].

To date, no treatment of particular significance has been identified for managing patients with multiple serous PEDs. Patients without signs of CNVM who retain good visual acuity can be monitored without the need for intervention.

## Conclusions

Our case underlines the importance of a meticulous clinical examination and the use of multimodal imaging. Moreover, it is highly recommended that every patient with multifocal idiopathic PEDs must undergo systemic investigations to rule out any potential systemic associations. Finally, patients’ awareness and vigilance for any warning signs should be paired with a long-term follow-up and monitoring for potential ophthalmic complications.
